# Inhibition of Sphingosine-1-Phosphate Receptor 2 Prevents Thoracic Aortic Dissection and Rupture

**DOI:** 10.3389/fcvm.2021.748486

**Published:** 2021-12-17

**Authors:** Guangwei Pan, Mengyang Liao, Yong Dai, Yang Li, Xiaole Yan, Wuqian Mai, Jinping Liu, Yuhua Liao, Zhihua Qiu, Zihua Zhou

**Affiliations:** ^1^Department of Cardiology, Union Hospital, Tongji Medical College, Huazhong University of Science and Technology, Wuhan, China; ^2^Institute of Cardiology, Union Hospital, Tongji Medical College, Huazhong University of Science and Technology, Wuhan, China; ^3^Key Lab of Molecular Biological Targeted Therapies of the Ministry of Education, Union Hospital, Tongji Medical College, Huazhong University of Science and Technology, Wuhan, China

**Keywords:** thoracic aortic dissection, S1PR2 inhibition, JTE013, inflammation, neutrophil extracellular traps

## Abstract

**Background:** Numerous pieces of evidence have indicated that thoracic aortic dissection (TAD) is an inflammatory disease. Sphingosine-1-phosphate receptor 2 (S1PR2) signaling is a driver in multiple inflammatory diseases. Here, we examined the S1PR2 expression in TAD lesions and explored the effect of interfering with S1PR2 on TAD formation and progression.

**Methods:** Aorta specimens and blood samples were collected from patients with TAD and matched controls. The expression of S1PR1, S1PR2, and S1PR3 was examined. The effect of inhibiting S1PR2 on TAD was evaluated in a TAD mouse model induced by β-aminopropionitrile fumarate (BAPN) and AngII. The presence of sphingosine kinase 1 (SPHK1), S1P, and neutrophil extracellular traps (NETs) was investigated. Further, the possible association between S1PR2 signaling and NETs in TAD was analyzed.

**Results:** In the aortic tissues of patients with TAD and a mouse model, the S1PR2 expression was significantly up-regulated. In the TAD mouse model, JTE013, a specific S1PR2 antagonist, not only blunted the TAD formation and aortic rupture, but also preserved the elastic fiber architecture, reduced the smooth muscle cells apoptosis level, and mitigated the aortic wall inflammation. Augmented tissue protein expression of SPHK1, citrullinated histone H3 (CitH3, a specific marker of NETs), and serum S1P, CitH3 were detected in TAD patients. Surgical repair normalized the serum S1P and CitH3 levels. Immunofluorescence staining revealed that S1PR2 colocalized with NETs. The protein expression levels of SPHK1 and serum S1P levels positively correlated with the protein expression and serum levels of CitH3, separately. Furthermore, JTE013 treatment reduced NETs accumulation.

**Conclusion:** Inhibiting S1PR2 attenuates TAD formation and prevents aortic rupture. Targeting S1PR2 may provide a promising treatment strategy against TAD.

## Introduction

Thoracic aortic dissection (TAD) is an emergent and serious cardiovascular disease that carries a high risk of mortality (1, 2). Despite significant improvements in surgical and endovascular repair, no pharmacologic therapy has been clinically confirmed to be effective in preventing the disease progression and reducing morbidity and mortality (2, 3). Therefore, there is an immediate necessity to discover an effective treatment strategy that prevents the disease progression.

The key feature of TAD is progressive aortic medial structure degeneration. Inappropriate inflammation has been confirmed as a central trigger to this degeneration, however, its accurate mechanisms have not yet been clearly clarified (1, 2). Sphingosine-1-phosphate (S1P), a bioactive sphingolipid, is generated from the metabolism of cell membrane sphingolipids mainly through sphingosine kinase (SPHK), and directly acts as an intracellular signaling molecule or natural ligand of different types of S1P receptors (S1PRs) to regulate diverse cellular functions (4–6). S1PRs participate in many essential physiological processes such as adaptive immune cell trafficking, vascular development, and homeostasis. Further, S1PR signaling also acts as a contributing factor in the autoimmune, inflammatory, neurological, cardiovascular, oncologic, and fibrotic diseases, which provides multiple promising targets to discover treatment strategies (6). S1PR1 has been conformed to exert a beneficial effect on the vasculature by improving the endothelial function, strengthening the endothelial barrier integrity, inhibiting inappropriate angiogenesis, and possessing anti-atherosclerotic properties; whereas S1PR2 often exerts the opposite cellular functions to S1PR1 (7, 8). In addition, S1PR2 signaling has been widely recognized as a driver of multiple inflammatory diseases, such as atherosclerosis, acute vascular inflammation, cerebral ischemia/reperfusion injury, rheumatoid arthritis, and hepatic inflammation (9–12). To date, the role of S1PR2 in TAD, and whether interfering with S1PR2 could attenuate TAD formation remain unknown.

Neutrophils are the predominant inflammatory cell type infiltrating the TAD aorta lesion, meanwhile, play a crucial role in the aortic wall inflammation involved in the TAD formation (13–15). Of note, neutrophil depletion by administrating anti-granulocyte-differentiation antigen-1 antibody inhibits TAD formation in a mouse model partly due to the reduction of matrix metalloproteinase (MMP) 9 activity (13), however, additional roles of neutrophil also seem to be involved in the TAD formation. The ability of neutrophils to release neutrophil extracellular traps (NETs) (16, 17), fibrous web-like structures comprised discharged chromatin, histones, and neutrophil granule proteins (18), attracts increasing interest due to its pro-inflammatory nature (17, 19). Apart from the early recognized antimicrobial effect (20), recent studies have revealed other vital roles of inappropriate released NETs in the pathogenesis process of diseases involving sterile inflammation, such as atherosclerosis, vasculitis, thrombosis formation, and abdominal aortic aneurysm (AAA) (16, 21–23). Interestingly, NETs inhibition could blunt the AAA formation in mice by mitigating the inflammation of the arterial wall (22, 23). However, whether NETs play a role in the TAD formation remains elusive. Of note, the S1PR2 pathway has been proven to participate in regulating neutrophil survival and that interference with the S1PR2 pathway elicits neutrophil apoptosis which subsequently mitigates acute inflammation induced by LPS *in vivo* (24). Furthermore, the prior study has indicated that S1PR2 signaling induced the redirection of neutrophils apoptosis to NETs formation *in vitro*. Inhibiting S1PR2 resulted in a restraint of NETs formation and decreased hepatic inflammation (25). Therefore, the association between S1PR2 signaling and NETs in the TAD formation and progression merits further investigation.

In this study, we sought to examine the expression of S1PR2 in the aortic tissues of patients with TAD and a mouse model, and explore the effect of JTE013, a specific small-molecule S1PR2 inhibitor that was shown to offer a potential means of treating various inflammatory conditions (9–12), on the TAD formation by using a TAD mouse model. Moreover, we investigated the presence of SPHK1, S1P, and NETs in patients with TAD and a mouse model. Finally, we analyzed the possible association between S1PR2 signaling and NETs in TAD.

## Materials and Methods

### Patient Enrollment and Sample Collection

This human study was conducted between August 2020 and February 2021 in Wuhan Union Hospital and was approved by the Medicine Ethics Committee of Wuhan Union Hospital, and adhered to the Declaration of Helsinki. All participants gave written informed consent before enrollment. Finally, 55 patients with sporadic TAD who were undergoing open-heart surgery for TAD after pre-operative CT were consecutively enrolled in this study; 67 volunteers from the Health Examination Center were enrolled as control after matching in sex, age, and body mass index (BMI) with the patients with TAD. The exclusion criteria included genetic syndrome related to aortic disease (Marfan syndrome, Ehlers-Danlos syndrome, Turner, Loeys–Dietz, etc.), aortic trauma, pseudo-aneurysm, previous aortic surgery, recent (<1 year) cancer, and systemic autoimmune or hematological disease (26, 27).

The serum and plasma samples collected from the patients with TAD and volunteers were divided into 0.5ml per centrifuge tube, then stored at −80°C. The fresh TAD specimens (*n* = 10) were routinely excised from the outer aortic wall of the false lumen (28) during aortic repair surgery, and control ascending aortic tissues without aortic aneurysm/dissection, collagen disease, or previous aortic repair were obtained from heart transplantation donors (*n* = 6). All specimens were rinsed with pre-cold 0.9% saline, fixed in 4% paraformaldehyde overnight, and then embedded in paraffin for histology analysis, or snap-frozen in liquid nitrogen and stored at −80°C for protein extraction.

### Animal Studies

All animal studies were approved by the Ethics Committee of Tongji Medical College of Huazhong University of Science and Technology. The male C57BL/6J mice were purchased from the Vital River Laboratory Animal Technology Company (Beijing, China), and housed in a specific pathogen-free laboratory at 22°C, with a 12-h light-dark cycle.

The TAD mouse model was induced as described previously (13, 14, 29). Briefly, 3-week-old male mice were fed on a regular diet and administered freshly prepared β-aminopropionitrile fumarate (BAPN) (A3134, Sigma-Aldrich, St Louis, MO, USA) dissolved in drinking water (1 g/kg/d) for 4 weeks. After administering 4-week BAPN, osmotic minipumps (1003D, Alzet, Palo Alto, CA, USA) filled with 1 μg/kg per minute AngII (ALX-151-039-M025, ENZO Biochem, Farmingdale, NY, USA) were subcutaneously implanted, and the mice were euthanized 24 h after implantation.

In this study, 3-week-old male C57BL/6J mice were randomly divided into three groups: (1) the CON (vehicle) group (*n* = 32, administered normal drinking water and vehicle); (2) the BAPN + AngII + vehicle group (*n* = 32, administered BAPN, AngII, and vehicle); (3) the BAPN + AngII + JTE013 group (*n* = 32, administered BAPN, AngII, and JTE013). The mice in group 3 were administrated with JTE013 (15 mg/kg, Cayman) by intraperitoneal (i.p.) injection for 28 days during BAPN administered, while mice in other groups were administered with an equal volume of vehicle (5% DMSO dissolved in PBS). The blood pressure (the tail-cuff method) and body weights were measured weekly in all groups.

The mice that died before the end of the experiment were autopsied immediately, and blood clots were found in the thoracic cavities of all dead mice. The other surviving mice were euthanized at the end of the experiment, the serum samples were collected for further analyses, and the aorta was harvested, then rinsed with pre-cold 0.9% saline to remove any residual blood in the lumen, fixed in 4% paraformaldehyde overnight and then embedded in paraffin for histology analysis, or snap-frozen in liquid nitrogen, then stored at −80°C for protein extraction.

The aortic rupture was defined as causing premature death, and adjacent body cavity containing hemorrhage (29, 30). The aortic dissection was characterized by the formation of a false lumen containing blood within the medial layer (31).

### Hematoxylin and Eosin Staining and Elastic Fiber Staining

Paraffin-embedded sections of aortic tissues (5 μm) were stained with hematoxylin and eosin and elastin-van gieson according to the manufacturer's instructions. The extent of elastic fiber fragmentation was analyzed on a scale of 1 to 4 (Grade 1, intact, well-organized elastic laminae; Grade 2, elastic laminae with some interruptions and breaks; Grade 3, severe elastin fragmentation or loss; and Grade 4, severe elastin degradation with visible ruptured sites) (28, 31, 32).

### Immunohistochemical Staining

Sections of aortic tissues (5 μm) were deparaffinized, rehydrated, antigen retrieved, and then blocked with normal blocking serum according to the previous method (33). The sections were then incubated with antibodies including S1PR2 (NBP2-26691, NOVUS, Littleton, CO, USA), S1PR1 (55133-1-AP, Proteintech, Wuhan, China), CD4 (553043, BD Biosciences, San Jose, CA, USA), CD8 (01041D, BD Biosciences, San Jose, CA, USA), CD31 (553370, BD Biosciences, San Jose, CA, USA), Mac-3 (553322, BD Biosciences, San Jose, CA, USA), and Myeloperoxidase (MPO) (ab208670, Abcam, Cambridge, MA, USA) overnight at 4°C, followed by staining with appropriate secondary antibodies. Finally, the sections were visualized with diaminobenzidine (DAB) and counterstained with hematoxylin. Immunohistochemical sections of human aortic tissues were scanned with an automatic scanning system to obtain the complete image of the entire specimen (NanoZoomer S360, Hamamatsu, Japan). The images presented in the figures were captured under low magnification field (10X) and high magnification field (40X) with an Olympus biological microscope (Japan) to maintain the consistent scale bar. All images of each target protein were obtained using identical conditions for the same period on the same day. CD4+ T-cells, CD8+ T-cells, CD31+ micro-vessels, and MPO+ neutrophils were counted and presented as numbers per aortic section. The relative S1PR2, S1PR1, and Mac-3+ macrophages contents within the aortas were quantified by measuring the immunostaining signal positive area using a computer-assisted image analysis software (Image-Pro Plus; Media Cybernetics, Bethesda, MD, USA). The data were analyzed in a blinded fashion, by two independent observers. Differences of the percent signal-positive area/sum total area of stain or positive cell numbers in different groups were compared.

### Immunofluorescence Staining

Sections of aortic tissues (5 μm) were deparaffinized and rehydrated, the antigen of sections was retrieved and then blocked with normal blocking serum according to the previous method (33). The sections were then incubated with antibodies against S1PR2 (NBP2-26691, NOVUS, Littleton, CO, USA), CitH3 (ab5103, Abcam, Cambridge, MA, USA), MPO (ab208670, Abcam, Cambridge, MA, USA) overnight at 4°C, followed by incubating with appropriate secondary antibodies, and then counterstained with 4,6-diamidino-2-phenylindole (DAPI) (Thermo Fisher Scientific, MA, USA) for nuclei. All immunofluorescence sections were scanned with an automatic scanning system (Pannoramic MIDI, 3DHISTECH, Hungary). The CitH3 and MPO double-positive area and the sum total area were quantified using ImageJ. Data were analyzed in a blinded fashion, by two independent observers. Differences of the percent double-positive area/sum total area of stain in different groups were compared.

### Terminal Deoxynucleotidyl Transferase dUTP Nick-End Labeling (TUNEL) Assay and Immunofluorescence Staining

TUNEL staining was performed according to the manufacturer's instructions by using a cell death detection kit (Roche Applied Science, Basel, Switzerland). In this study, TUNEL and immunofluorescence co-staining was performed as follows: paraffin-embedded slides were permeabilized and subjected to TUNEL staining, the sections were then blocked and stained with an α-SMA (ab7817, Abcam, Cambridge, MA, USA) antibody overnight at 4°C, followed by incubating with the appropriate secondary antibody. All sections were scanned with an automatic scanning system (Pannoramic MIDI, 3DHISTECH, Hungary). The number of TUNEL positive cells and the number of total cell nuclei were quantified using ImageJ, and the percentage of positive cells was calculated for comparison in different groups. Data were analyzed by two independent observers who were blinded to the sources of samples.

### Western Blotting

The protein extracted from the aortic tissue was separated on a sodium dodecyl sulfate–polyacrylamide gel electrophoresis (SDS-PAGE) gel and transferred to polyvinylidene difluoride membranes. The membranes were blocked with 5% skim milk, then incubated overnight at 4°C with the primary antibodies against S1PR2 (NBP2-26691, NOVUS, Littleton, CO, USA), S1PR1 (55133-1-AP, Proteintech, Wuhan, China), SPHK1 (10670-1-AP, Proteintech, Wuhan, China), CitH3 (ab5103, Abcam, Cambridge, MA, USA), Arg1 (16001-1-AP, Proteintech, Wuhan, China), CD206 (18704-1-AP, Proteintech, Wuhan, China), iNOS (18985-1-AP, Proteintech, Wuhan, China), MCP1 (ab7202, Abcam, Cambridge, MA, USA), and TNFα (ab1793, Abcam, Cambridge, MA, USA); followed by incubating with the appropriate secondary antibody. Finally, the protein bands were visualized using Clarity Enhanced Chemiluminescence (Bio-Rad Laboratories, CA, USA) after the membranes were washed.

### Real-Time Quantitative Polymerase Chain Reaction (RT-qPCR) Analysis

The total RNA from the aortic tissue was extracted using RNAiso Plus (Takara Bio, Shiga, Japan) following the manufacturer's protocol, and transcribed to complementary DNA (cDNA) using the MultiScribe® Reverse Transcriptase (Thermo Fisher Scientific). RT-qPCR was performed on a StepOne Real-Time PCR system (Applied Biosystems, CA, USA) using a TB Green Premix Ex Taq (Takara, Shiga, Japan). The β-actin expression was quantified and used as the endogenous control.

### Laboratory Investigations

Human D-Dimer, hs-CRP, lipid profiles as well as complete blood cell and platelet count were assayed by routine laboratory techniques using blinded quality control specimens in the department of the biochemical laboratory at Wuhan Union Hospital.

The concentration of human and mouse serum S1P, CitH3 were measured by the S1P ELISA kit (ELK8273, ELK biotech, Wuhan, China) and CitH3 ELISA kit (501620, Cayman, Ann Arbor, MI, USA) according to the manufacturer's instructions.

### Statistical Analysis

The results were expressed as mean ± SD or median and interquartile range for continuous variables, and as total number (percent frequency) for categorical variables. Shapiro-Wilk test was used to determine the data distribution normality. Differences between two groups of data with normally distributed variables were compared with unpaired two-tailed *t*-test, while the Mann-Whitney test was used for abnormally distributed variables. Multigroup comparisons (≥3 groups) were performed using the one-way ANOVA test followed by Bonferroni's *post-hoc* test for equal variance or Games–Howell's *post-hoc* test for unequal variance. The Kruskal–Wallis test was applied for multigroup comparisons (≥3 groups) with abnormally distributed variables. The categorical variables in this study were compared using the chi-square test, the Fisher's exact test was applied when appropriate. Pearson correlation test was used to evaluate the association between S1PR2 signaling and NETs. A two-tailed probability level of <0.05 was considered statistically significant. All statistics were performed by using IBM SPSS (version 23.0, Chicago, IL, USA) and GraphPad Prism (version 7.0, San Diego, CA, USA).

## Results

### Clinical Characteristics of the Patients Included in the Study

The clinical characteristics of the control and patients with TAD who participated in the study are shown in Tables 1, 2. Among the participants that provided aorta specimens, no significant difference was observed in age, sex, smoking history, BMI, and comorbidities (Table 1). Among the participants that provided blood samples, there was no significant difference in smoking history, heart rate, lipid profiles, fasting plasma glucose (FPG), platelet, Left ventricular ejection fraction (LVEF), and the rate of diabetes, hyperlipidemia, extracardiac arteriopathy in the two groups. Compared with the matched control, significantly elevated systolic blood pressure (SBP), diastolic blood pressure (DBP), hypersensitive C-reactive protein (hs-CRP), D-Dimer, aortic diameter, white blood cell (WBC), neutrophils, monocytes count, and decreased lymphocytes count were detected in patients with TAD (Table 2).

**Table 1:** Clinical characteristics of patients provided aorta specimens.

	**Controls (*n* = 6)**	**TAD (*n* = 10)**	***P*** **-value**
Age (years)	46.8 ± 6.3	53.4 ± 8.3	0.119
Man (*n*, %)	4 (66.7)	8 (80.0)	0.604
Smoking history (*n*, %)	2 (33.3)	5 (50.0)	0.633
BMI (kg/m^2^)	22.5 ± 1.3	23.5 ± 2.7	0.412
Hypertension (*n*, %)	1 (16.7)	6 (60.0)	0.121
Diabetes (*n*, %)	0 (0)	2 (20.0)	0.500
Hyperlipidemia (*n*, %)	1 (16.7)	3 (30.0)	0.511
Extracardiac arteriopathy (*n*, %)	0 (0)	3 (30.0)	0.214

*TAD, thoracic aortic dissection; BMI, body mass index. Data are expressed as means ± SD, or number (%)*.

**Table 2:** Clinical characteristics of patients provided blood sample.

	**Controls (*n* = 55)**	**TAD (*n* = 67)**	***P*** **-value**
Age (years)	52.7 ± 12.5	53.4 ± 13.1	0.772
Man (*n*, %)	45 (81.8)	56 (83.6)	0.797
Smoking history (*n*, %)	20 (36.4)	30 (44.8)	0.347
BMI (kg/m^2^)	24.2 ± 2.3	24.9 ± 3.5	0.261
Systolic pressure (mmHg)	116 ± 12	139 ± 20	<0.001
Diastolic pressure (mmHg)	73 ± 9	81 ± 15	<0.001
Heart rate	80.6 ± 14.3	81.4 ± 15.1	0.762
Hypertension (*n*, %)	6 (10.9)	44 (65.7)	<0.001
Diabetes (*n*, %)	2 (3.64)	3 (4.48)	0.816
Hyperlipidemia (*n*, %)	8 (14.6)	8 (11.9)	0.671
Extracardiac arteriopathy (*n*, %)	12 (21.8)	21 (31.3)	0.239
TG (mmol/L)	1.27 ± 0.81	1.37 ± 0.44	0.415
TC (mmol/L)	4.22 ± 0.75	4.24 ± 0.91	0.902
LDL-C (mmol/L)	2.52 ± 0.66	2.65 ± 0.77	0.353
HDL-C (mmol/L)	1.17 ± 0.28	1.10 ± 0.29	0.186
FPG (mmol/L)	5.25 ± 1.04	5.63 ± 1.14	0.058
hs-CRP (mmol/L)	1.33 ± 1.17	9.17 ± 3.31	<0.001
D-Dimer (μg/mL)	0.16 (0.12–0.22)	1.04 (0.73–1.31)	<0.001
Diameter (mm)	29.9 ± 2.8	43.5 ± 5.8	<0.001
LVEF (%)	62.1 ± 4.8	62.6 ± 3.8	0.525
WBC (×10^9^/L)	6.21 ± 1.59	10.4 ± 4.53	<0.001
Neutrophils (×10^9^/L)	3.91 ± 1.33	8.48 ± 4.17	<0.001
Lymphocytes (×10^9^/L)	1.49 ± 0.61	1.06 ± 0.41	<0.001
Monocytes (×10^9^/L)	0.46 ± 0.25	0.73 ± 0.35	<0.001
Platelet (g/L)	200 ± 56	183 ± 78	0.163

*TAD, thoracic aortic dissection; BMI, body mass index; SBP, systolic blood pressure; DBP, diastolic blood pressure; FPG, fasting plasma glucose; TG, triglycerides; TC, total cholesterol; LDL-C, low-density lipoprotein cholesterol; HDL-C, high-density lipoprotein cholesterol; WBC, white blood cell. Data are expressed as means ± SD, median (interquartile range), or number (%)*.

### Increased S1PR2 Accumulation in Human and Murine TAD Lesions

**Figure 1:**
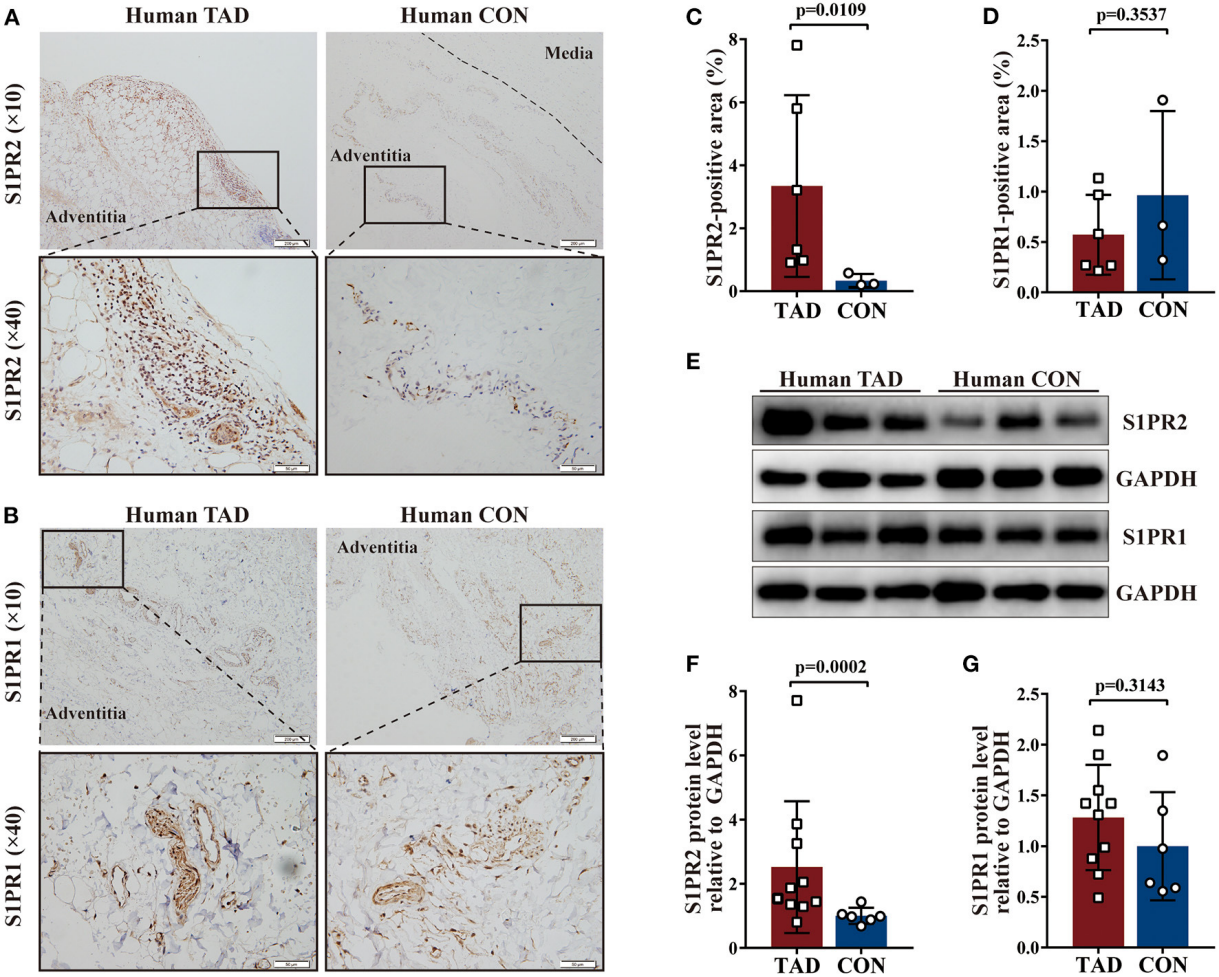
Increased Sphingosine-1-phosphate receptor 2 (S1PR2) accumulation in human thoracic aortic dissection (TAD) lesions. Representative immunohistochemistry images and the corresponding analysis of human S1PR2 **(A,C)** and S1PR1 **(B,D)** in TAD lesions (*n* = 6) and normal thoracic aorta (*n* = 3). Representative immunoblot and the corresponding analysis of human S1PR2 **(E,F)** and S1PR1 **(E,G)** in TAD lesions (*n* = 10) and normal thoracic aorta (*n* = 6). Scale: 200 μm, inset: 50 μm. Data are expressed as means ± SD.

Firstly, the expression of S1PR1, S1PR2, and S1PR3 was detected in the aortic tissues of patients with TAD. Immunohistochemical staining showed that S1PR2 and S1PR1 mainly expressed in the adventitia of the aortic wall probably due to their relatively high expression in the immune cells, and revealed that S1PR2 was highly expressed in the aortic tissues of patients with TAD when compared with control aortic tissues, while no difference of S1PR1 expression was observed in the two groups (Figures 1A–D). The western blot analysis (Figures 1E–G) and RT-qPCR experiments (Supplementary Figures 1A,B) revealed the same results, meanwhile, found that the expression of S1PR3 also showed no difference in the two human groups (Supplementary Figures 1C,G).

**Figure 2:**
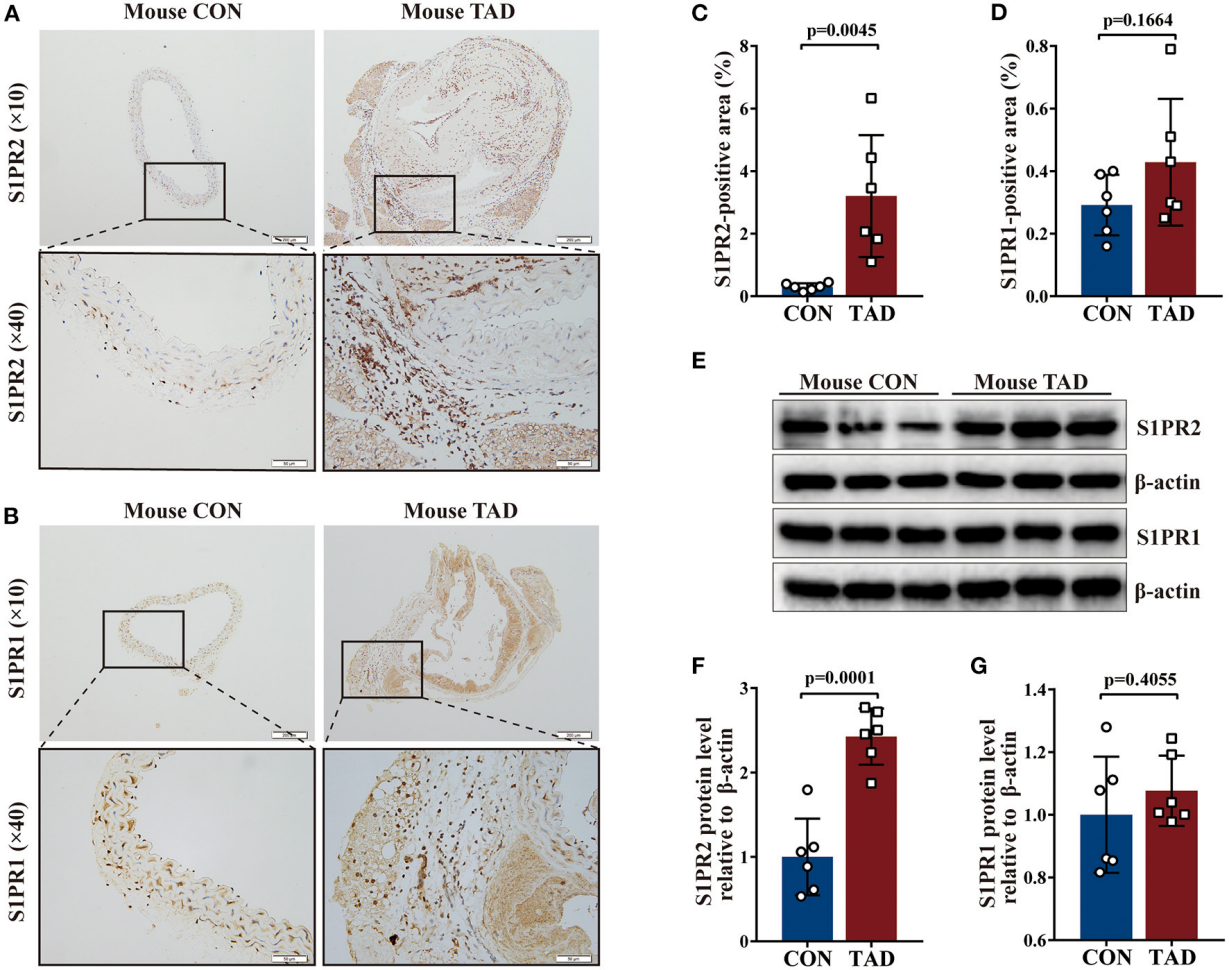
Increased S1PR2 accumulation in mouse TAD lesions. Representative immunohistochemistry images and the corresponding analysis of mouse S1PR2 **(A,C)** and S1PR1 **(B,D)** in TAD lesions (*n* = 6) and control thoracic aorta (*n* = 6). Representative immunoblot and the corresponding analysis of mouse S1PR2 **(E,F)** and S1PR1 **(E,G)** in TAD lesions (*n* = 6) and control thoracic aorta (*n* = 6). Scale: 200 μm, inset: 50 μm. Data are expressed as means ± SD.

Following human tissue studies, S1PR2 was also significantly up-regulated in the aortic tissues of TAD mouse, while the expression of S1PR1 and S1PR3 showed no difference between aortic tissues of TAD mouse and control mouse by using the same methods as for human tissue studies (Figures 2A–G and Supplementary Figures 1D–F,H). These results suggested that S1PR2 may participate in the TAD formation.

### Inhibition of S1PR2 by JTE013 Attenuated TAD Formation and Rupture in Mice

**Figure 3:**
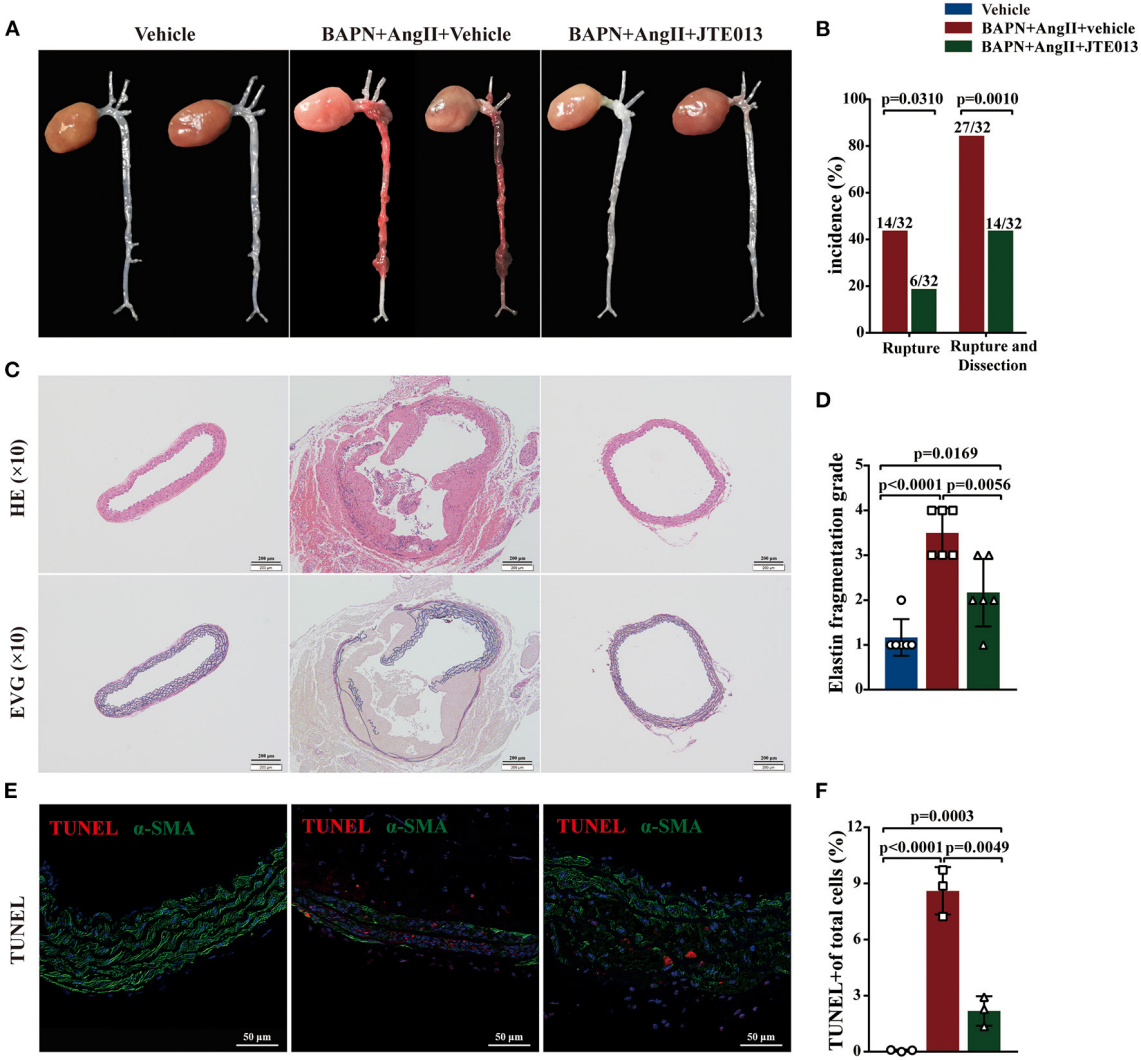
Inhibition of S1PR2 by JTE013 blunted acute aortic dissection (AAD) formation and prevent aortic rupture. **(A)** Representative morphology of the aortas in each group. **(B)** The JTE013 treatment attenuated AAD formation and prevent aortic rupture. **(C)** Representative images of hematoxylin and eosin staining and elastin-van gieson elastin staining of aorta lesions. **(D)** Elastic fiber fragmentation grade analysis (*n* = 6). **(E)** Representative images of Terminal deoxynucleotidyl transferase dUTP nick-end labeling (TUNEL) (red) and α-SMA (green) double-immunofluorescence staining area. **(F)** JTE013 treatment resulted in a restraint of SMCs apoptosis level (*n* = 3). Scale: 200 μm or 50 μm. Data are expressed as means ± SD.

During the 4-week animal experiments, there was no difference in body weight and systolic blood pressure measured before AngII administration between all experimental groups (Supplementary Figure 2). We found that BAPN+AngII administration induced aortic dissection not only mainly in the thoracic aortas but also in the abdominal aortas or even the carotid artery (Figure 3A). Importantly, treating the BAPN+AngII challenged mice with JTE013 blunted TAD formation compared with those mice treated with the vehicle, and also diminished the incidence of aortic rupture (Figures 3A,B). In the meantime, the JTE013 treatment preserved the elastic fiber architecture and reduced elastic fiber fragmentation and resulted in a restraint of smooth muscle cells (SMCs) apoptosis level (Figures 3C–F).

### Inhibition of S1PR2 by JTE013 Reduced Inflammatory Infiltration and Angiogenesis

**Figure 4:**
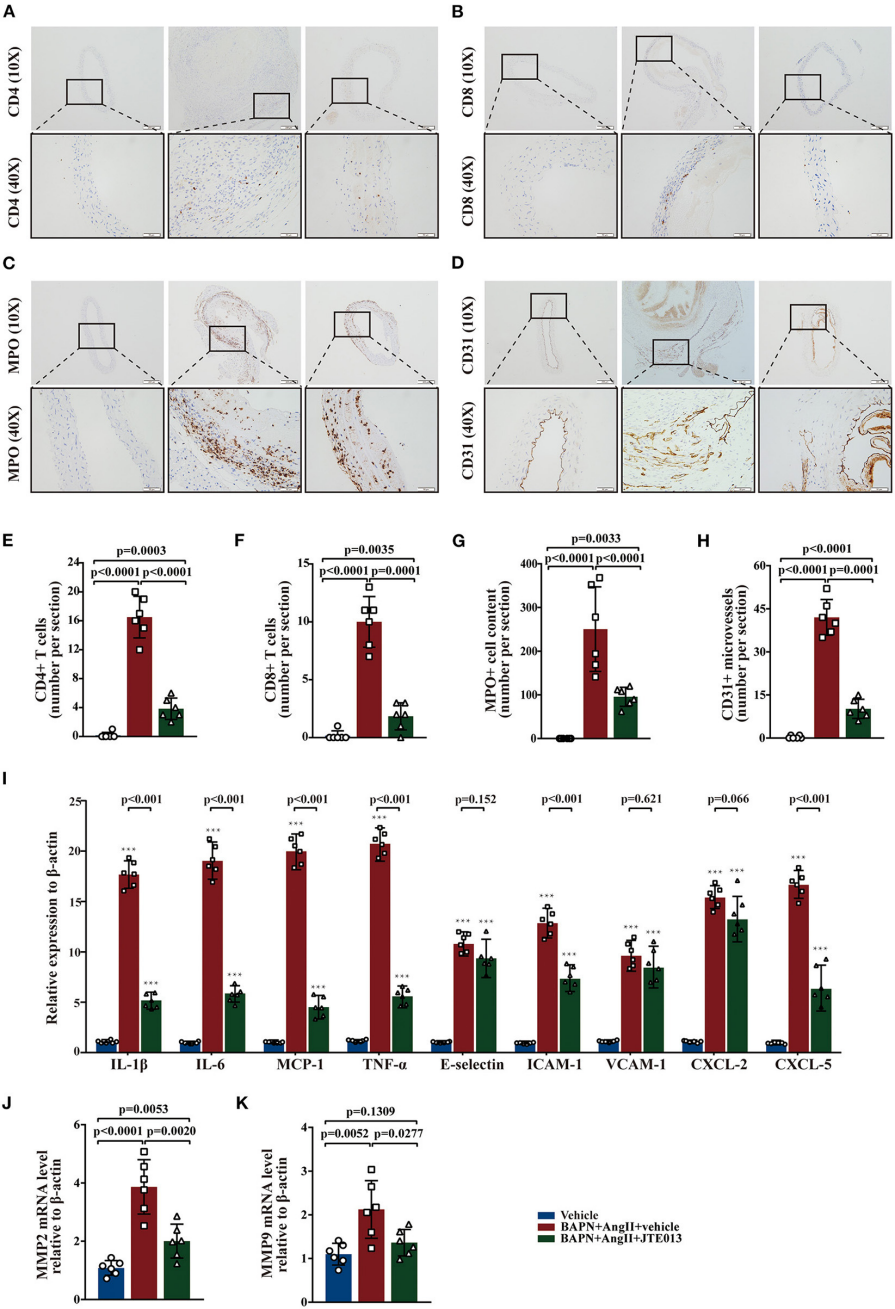
Inhibition of S1PR2 by JTE013 reduced inflammatory infiltration and angiogenesis. **(A,E)** Lesion CD4+ T-cell numbers and corresponding analysis. **(B,F)** Lesion CD8+ T-cell numbers and corresponding analysis. **(C,G)** Lesion MPO+ neutrophil numbers and corresponding analysis. **(D,H)** Lesion CD31+ micro-vessel numbers and corresponding analysis. **(I)** RT-qPCR determined lesion mRNA levels of IL-1β, IL-6, MCP-1, TNF-α, E-selectin, ICAM-1, VCAM-1, CXCL-2, and CXCL-5. **(J,K)** Analysis of MMP2 and MMP9 mRNA expression. *n* = 6 mice per group. ***p < 0.001 vs. the vehicle group. Scale: 200 μm, inset: 50 μm. Data are expressed as means ± SD. Myeloperoxidase (MPO), a marker for neutrophil abundance.

Several studies have shown that aortic wall inflammation and angiogenesis participate in TAD formation (1, 2). Further, we evaluated the effect of the JTE013-treatment on inflammatory infiltration and angiogenesis in the TAD mouse model. The immunohistochemistry assessment of aorta sections revealed a significant reduction in the numbers of CD4+ T-cells, CD8+ T-cells, MPO+ neutrophils, and CD31+ micro-vessels in the JTE013-treatment group compared with those mice treated with vehicle (Figures 4A–H).

Further, we performed RT-qPCR to measure the messenger RNA (mRNA) expression levels of multiple genes involved in inflammation, the JTE013-treated mice had significantly decreased mRNA expression of IL-1β, IL-6, MCP-1, TNF-α, ICAM-1, and CXCL-5 compared with the vehicle-administrated mice (Figure 4I). Meanwhile, the JTE013 treatment led to a restraint of the mRNA expressions of MMP2 and MMP9 (Figures 4J,K).

### Inhibition of S1PR2 by JTE013 Diminished Macrophages Accumulation and the Hallmarks of M1-Like Macrophage

**Figure 5:**
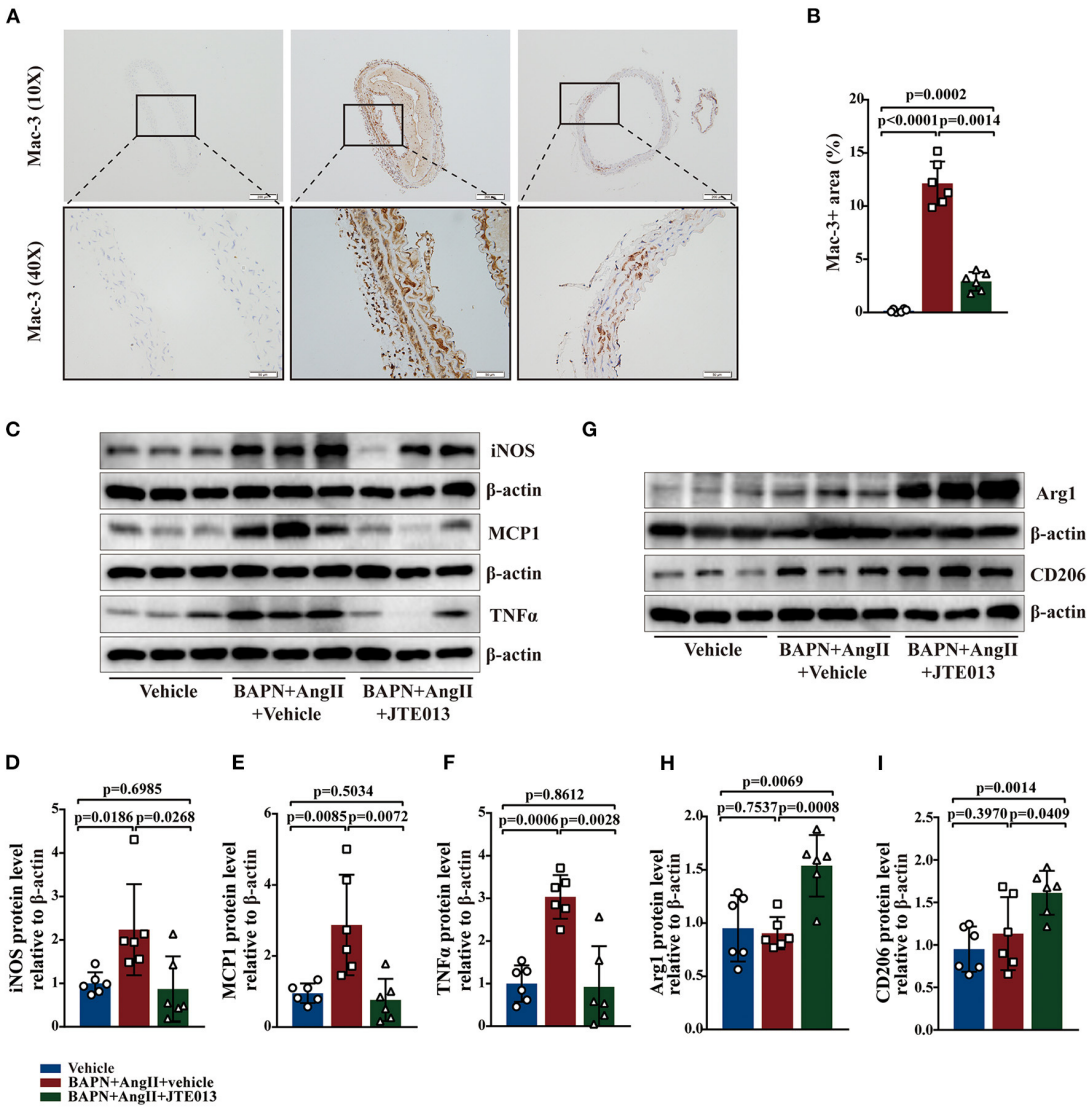
Inhibition of S1PR2 by JTE013 diminished macrophages accumulation and the hallmarks of M1 macrophage. **(A,B)** Lesion Mac-3+ area and corresponding analysis (*n* = 6). **(C–F)** Representative immunoblot and the corresponding analysis of mouse iNOS, MCP1, and TNFα in each group (*n* = 6). **(G–I)** Representative immunoblot and the corresponding analysis of mouse Arg1 and CD206 (*n* = 6). Scale: 200 μm, inset: 50 μm. Data are expressed as means ± SD.

Macrophages play an important role in the process of TAD (34). The effect of JTE013 treatment on inappropriate macrophage inflammation was investigated in the aortic tissues of the TAD mouse model. In our study, the reduced lesion accumulation of macrophages was detected in the aortic sections of JTE013-treated mice (Figures 5A,B). Next, we examined the protein expression of M1-like and M2-like macrophage hallmarks. Treating the BAPN+AngII administrated mice with JTE013 resulted in a restraint of M1 markers expression including iNOS, TNF-α, and MCP-1 (Figures 5C–F), and an augmented M2 markers expression of Arg-1 and CD206 (Figures 5G–I). These results suggested that inhibiting S1PR2 with JTE013 may mitigate inappropriate macrophage inflammation in the aortic wall.

### Inhibition of S1PR2 by JTE013 Reduced NETs Accumulation

**Figure 6:**
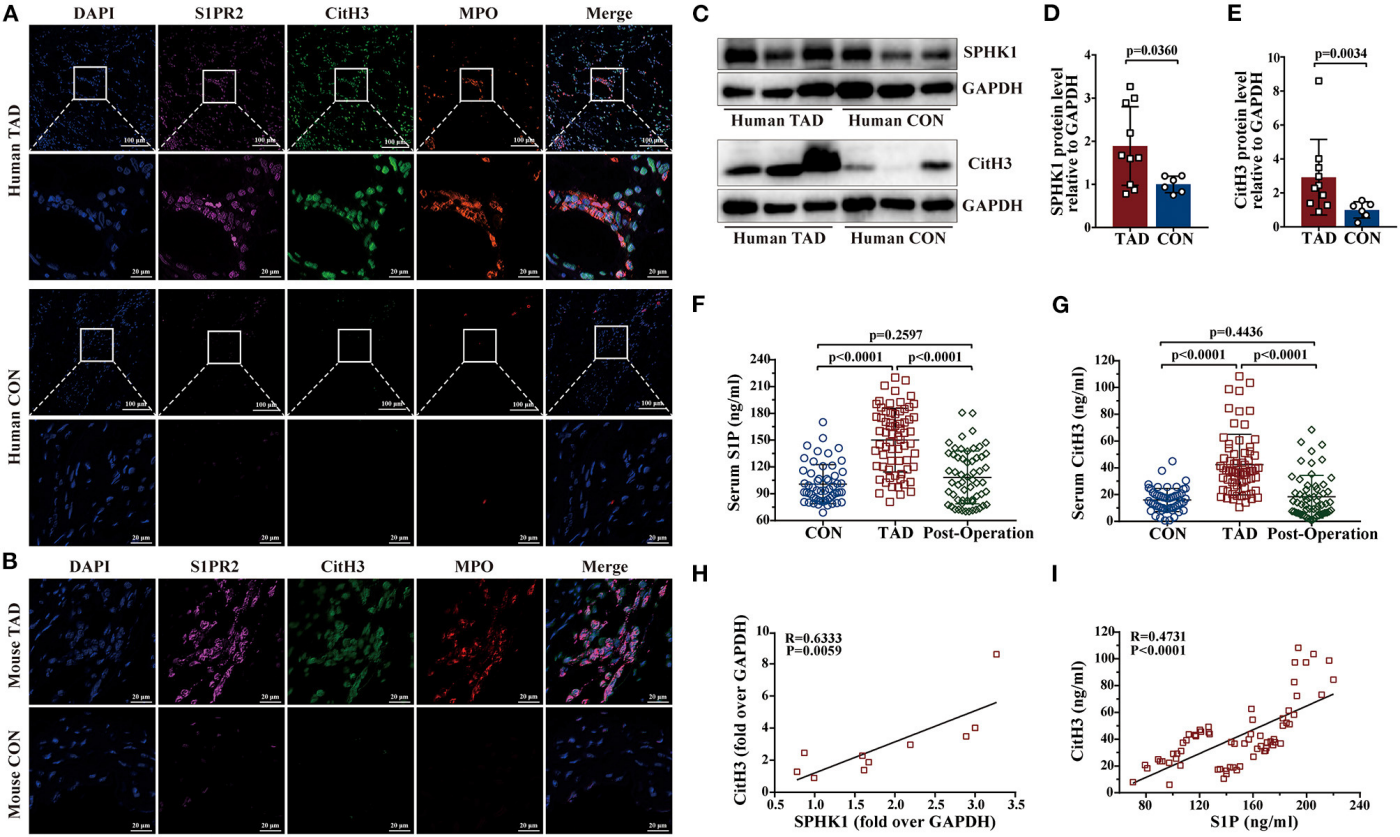
Activated S1P/S1PR2 signaling and neutrophil extracellular traps (NETs) had a positive correlation. **(A,B)** Immunofluorescent staining showing the colocalization of S1PR2 and NETs in human and mouse TAD lesions. **(C–E)** Representative immunoblot and the corresponding analysis of human SPHK1 and CitH3 in TAD lesions (*n* = 10) and normal thoracic aorta (*n* = 6). **(F,G)** Serum S1P and CitH3 levels in the matched control (*n* = 55), patients with TAD before surgery (*n* = 67) and TAD patients after surgery (*n* = 58). **(H)** SPHK1 protein level positively correlated with CitH3 protein level in patients with TAD. **(I)** Serum S1P level positively correlated with serum CitH3 level in patients with TAD. Scale: 100 μm or 20 μm. Data are expressed as means ± SD. CitH3, a specific marker of NETs; Myeloperoxidase (MPO), a marker for neutrophil abundance.

Prior studies have demonstrated that the S1P/S1PR2 pathway prolonged neutrophil longevity (24), and induced the redirection of neutrophil apoptosis to NETs formation *in vitro* (25). In our study, immunofluorescence staining revealed that S1PR2 colocalized with NETs in both human and murine TAD lesions (Figures 6A,B). Elevated protein expressions of SPHK1 (a rate-limiting enzyme in S1P generation) and citrullinated histone H3 (CitH3, a specific marker of NETs) were detected in human TAD lesions (Figures 6C–E). Moreover, Serum S1P and Cith3 levels were significantly higher in the human TAD group compared with the control, while after surgical treatment, the serum levels of S1P and CitH3 were both diminished in patients with TAD (Figures 6F,G). These findings showed that the S1P/S1PR2 pathway and NETs may simultaneously participate in the TAD formation. Meanwhile, the correlation analysis revealed that the protein expression of SPHK1 and serum S1P levels positively correlated with protein expression and serum levels of CitH3, separately (Figures 6H,I). The above-mentioned results suggested a potential correlation between activated S1P/S1PR2 signaling and NETs density in human and murine TAD.

**Figure 7:**
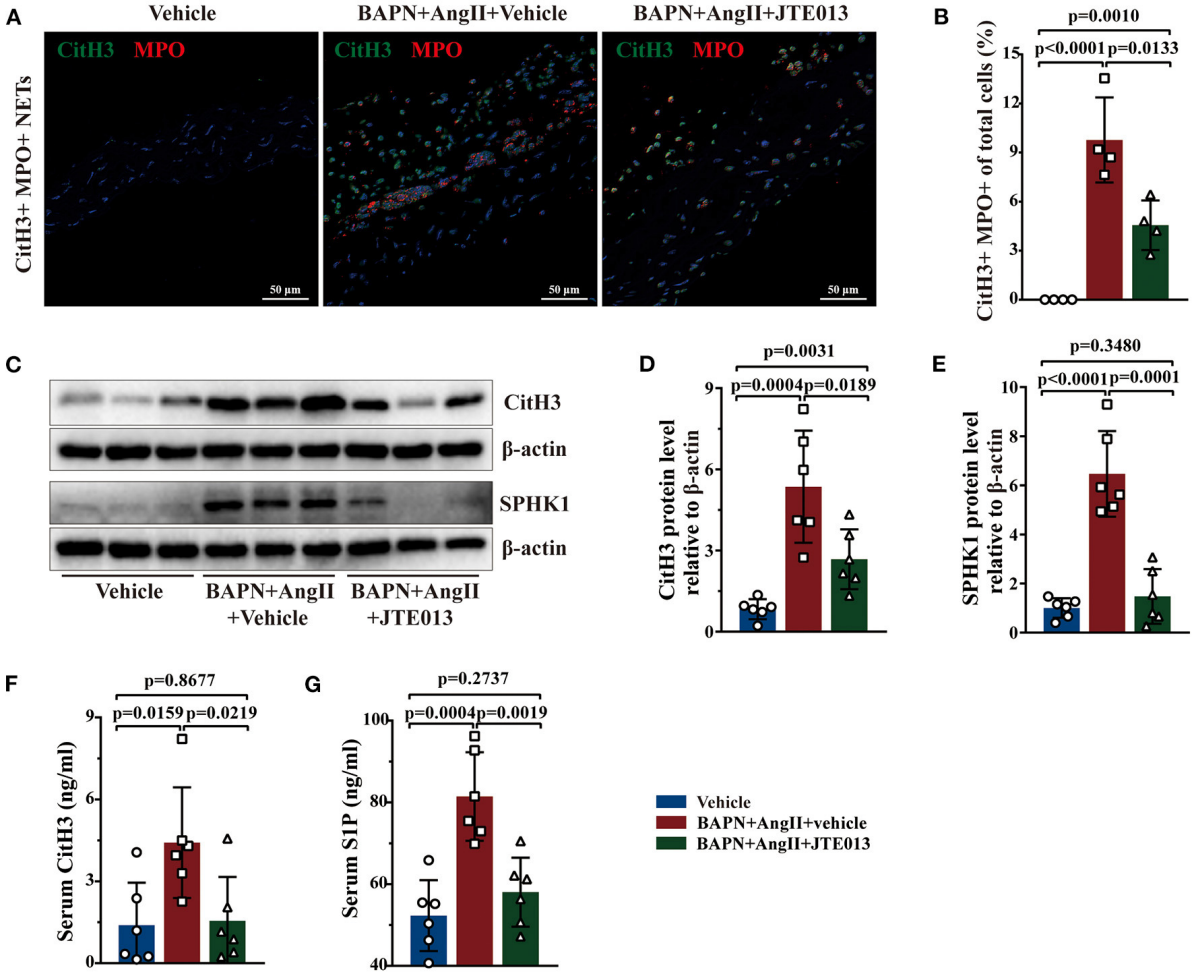
Inhibition of S1PR2 by JTE013 reduced NETs accumulation. **(A)** Representative images of CitH3+ (green) MPO+ (red) double-immunofluorescence staining NETs area in mouse TAD lesions from each group. **(B)** The JTE013 treatment diminished NETs density (*n* = 4). **(C–E)** Representative immunoblot and the corresponding analysis of mouse CitH3 and SPHK1 in each group (*n* = 6). **(F,G)** JTE013 treatment reduced serum CitH3 and S1P levels (*n* = 6). Scale: 50 μm. Data are expressed as means ± SD. CitH3, a specific marker of NETs; MPO, a marker for neutrophil abundance.

Further, immunofluorescent double staining revealed the reduction of CitH3+ MPO+ NETs accumulation in the aorta lesions of mice treated with JTE013 when compared with the vehicle-administrated mice (Figures 7A,B). Consistent with the immunofluorescent findings, diminished CitH3 protein expression was detected in murine aortas of the JTE013-treated group (Figures 7C,D). The ELISA yielded similar results, JTE013 treatment led to a restraint of circulating CitH3 levels (Figure 7F). In the meantime, the SPHK1 protein expression and the serum S1P level were reduced after the JTE013 treatment compared with those mice treated with vehicle (Figures 7C,E,G) which is in line with human findings.

## Discussion

In this study, elevated S1PR2 expression was detected in patients with TAD and a mouse model. The JTE013 treatment not only significantly blunted the TAD formation and aortic rupture, but also preserved the elastic fiber architecture, reduced the elastic fiber fragmentation and SMCs apoptosis level, and mitigated the inflammation and angiogenesis in the aortic wall. In addition, we found a potential correlation between activated S1P/S1PR2 signaling and NETs density in patients with TAD and observed the restraint of NETs accumulation in the aortic tissues of JTE013 treatment mice. Therefore, inhibition of S1PR2 may provide a novel therapeutic approach for TAD.

Sphingosine-1-phosphate (S1P) acts as a crucial mediator of various vascular physiological and pathological processes (6, 7). Elevated S1P has been proven to correlate with stroke, heart failure, atherosclerosis, and hypertension in experimental studies (35–38). Moreover, a recent study revealed that S1P could increase various markers of inflammation and vascular dysfunction (39). S1P exerts its effect on vascular processes mainly by interacting with S1PR1-3 (6, 7). As mentioned earlier, S1PR1 exerts a particularly beneficial role in the vasculature, while S1PR2 often displays the cellular functions which are opposite to S1PR1 (7, 8). Although S1PR3 is recognized to play a positive role in neovascularization, the effect of S1PR3 on endothelial barrier integrity and atherosclerosis remains elusive. Moreover, S1PR3 plays a significant role in protecting the heart against ischemia/reperfusion injury (40). S1PR4 showed limited expression in the lymphatic system, S1PR5 is characteristic of the immune and nervous systems, while the two S1PRs have relatively low expression levels compared to S1PR1-3 (6–8). It is worthy of note that S1PR2 signaling has been confirmed to play a pathogenic role in a range of inflammation-related diseases, interestingly, atherosclerosis, acute vascular inflammation, vasculitis, and cerebral ischemia/reperfusion injury are included (9–12). Inappropriate inflammation has been confirmed as a key contributing factor to TAD. In our study, augmented serum S1P was detected in the patients with TAD and mouse model which is in line with the above-mentioned cardiovascular conditions (35–38). Additionally, S1PR2 expression was significantly up-regulated in the aortic tissues of patients with TAD and a mouse model. These results suggested that activated S1P/S1PR2 signaling may participate in the TAD formation.

Focusing on the role of S1PR2 played in the various vascular inflammatory conditions and the elevated S1PR2 expression detected in TAD aortic tissues, we explored the effect of S1PR2 inhibition on the TAD formation using a previously developed TAD mouse model (13, 14, 29). In this study, we found that treatment with JTE013, a specific S1PR2 inhibitor, attenuated TAD formation and aortic rupture in the mouse model of TAD. Besides, JTE013 treatment resulted in a restrain of the elastic fiber fragmentation and SMCs apoptosis level. In addition, CD4+ T-cells, CD8+ T-cells, MPO+ neutrophils were less accumulated in the aorta lesions of the JTE013-treated mice compared with the vehicle-treated mice. Moreover, the JTE013 treatment led to a restraint of the mRNA expression of the inflammatory cytokines including IL-1β, IL-6, MCP-1, TNF-α, ICAM-1, and CXCL-5, a neutrophil chemotactic chemokine that in charge of neutrophil recruitment to the inflammation lesion from circulation (41–44). Macrophages also play a vital role in the inflammatory process that contributes to TAD (34). The M1-like macrophage could cause arterial SMCs apoptosis by activating the tumor necrosis factor (TNF) and nitric oxide signaling pathways, moreover, macrophages also secrete proinflammatory cytokines and MMPs, which all act as vital accomplices in the TAD formation (45). While the M2-like macrophage may participate in the repair process of TAD remodeling (34). In our study, inhibiting S1PR2 with JTE013 reduced the lesion accumulation of macrophages, meanwhile, decreased the expression of the M1 marker including iNOS, TNF-α, and MCP-1, and also enhanced M2 markers expression including Arg-1 and CD206, which may indicate that JTE013 treatment mitigated inappropriate macrophages inflammation in the aortic wall. The above-mentioned results suggested that inhibiting S1PR2 with JTE013 attenuated TAD formation and prevented aortic rupture by promoting inflammation resolution in the aortic wall.

Neutrophils are the predominant leukocyte type in human peripheral blood and infiltrate into the aortic adventitia to play a causative role in the TAD formation (13–15). Previous studies showed that S1P could enhance the chemotaxis of neutrophils (46–49). S1P/S1PR2 signaling has been proven to prolong neutrophil longevity in multiple inflammatory conditions by limiting neutrophil apoptosis and that blockade of S1P synthesis or interference with the S1PR2 pathway mitigates acute inflammation induced by LPS *in vivo* (24). In this study, immunofluorescence staining revealed that S1PR2 colocalized with MPO (a marker for neutrophil abundance, Figures 6A,B) in the aortic tissues of patients with TAD and the mouse model, while the JTE013 treatment reduced the neutrophil accumulation in the aortic wall of TAD mouse. These findings indicated that activated S1PR2 signaling may participate in neutrophilic inflammation in the TAD formation.

Although neutrophil-derived MMPs have been confirmed to contribute to the pathogenicity of neutrophils in TAD development, the complete mechanisms of which remain unclear. NETs, a form of neutrophil activation, not only comprise a crucial part of the neutrophil-mediated defense mechanism but also act as an accomplice in sterile inflammation which seems deleterious to the progression of a range of diseases (16, 21–23). Further, NETs have been proven to play a pathogenic role in AAA formation (22, 23). In our study, elevated plasm neutrophils were detected in patients with TAD. Further, we observed a massive presence of NETs in the aortic tissues, serum of TAD patients, and mouse model. These results suggested that NETs may participate in TAD formation.

The S1P/S1PR2 pathway has been confirmed to correlate with NET formation and mediates the redirection of neutrophils apoptosis to the NET formation, S1PR2 blockade could reduce the NET formation and then alleviate hepatic inflammation (25). Based on the above-mentioned results, activated S1P/S1PR2 signaling may participate in regulating neutrophil fate and subsequent NETs formation. In our study, activated S1P/S1PR2 pathway and NETs were detected both in human and mouse TAD, and had a potential correlation following the prior study (25). Using a TAD mouse model, we found that inhibition of S1PR2 with JTE013 diminished the hallmarks of NETs including CitH3 protein expression, circulating CitH3 and MPO+ CitH3+ immunofluorescent co-staining area. Based on these findings, we revealed that the S1P/S1PR2 pathway may display a deleterious effect on TAD formation by promoting NETs formation. However, future studies are required to explore how S1P/S1PR2 signaling contributes to NETs formation by using mouse bone marrow neutrophils. Furthermore, mice with conditional deletion of S1PR2 in neutrophils could be valuable to deeply explore the relevant mechanisms in the TAD formation and progression.

Progressive vascular SMCs loss is a key event in the TAD formation and progression. In a recent study, TUNEL staining and the flow cytometry analysis of Annexin V-FITC/PI staining demonstrated that NETs stimulation increased the apoptosis of SMCs (50). Meanwhile, the results of another study showed that histone H4, one component of NETs, mediated membrane lysis of SMCs, which triggered arterial tissue damage and inflammation (51, 52). Besides, the proteases hanging on NETs fibers like MMPs could directly damage the aortic wall after being released (53). Based on these results, NETs may exert a damaging effect on the aortic wall in a variety of ways which may contribute to the pathogenesis of TAD. Further studies are needed to explore the exact underlying mechanisms.

In summary, our study demonstrates that S1PR2 signaling acts as a crucial accomplice in the TAD formation. Inhibiting S1PR2 by JTE013 alleviates TAD formation and aortic rupture. Moreover, JTE013 treatment leads to a restraint of SMCs apoptosis level, mitigates inflammation and angiogenesis in the aortic wall, and reduces NET accumulation. These findings suggest that inhibition of S1PR2 may provide a novel treatment strategy against TAD.

## Data Availability Statement

The original contributions presented in the study are included in the article/Supplementary Material, further inquiries can be directed to the corresponding author/s.

## Ethics Statement

The studies involving human participants were reviewed and approved by Medicine Ethics Committee of Wuhan Union Hospital. The patients/participants provided their written informed consent to participate in this study. The animal study was reviewed and approved by Ethics Committee of Tongji Medical College of Huazhong University of Science and Technology.

## Author Contributions

GP, ML, and ZQ conceived the hypothesis and designed the study. GP wrote and revised the paper. YaL, YD, XY, WM, and GP performed the research. YuL and JL analyzed the data. ZQ and ZZ supervised the research. All authors contributed to the article and approved the submitted version.

## Conflict of Interest

The authors declare that the research was conducted in the absence of any commercial or financial relationships that could be construed as a potential conflict of interest.

## Publisher's Note

All claims expressed in this article are solely those of the authors and do not necessarily represent those of their affiliated organizations, or those of the publisher, the editors and the reviewers. Any product that may be evaluated in this article, or claim that may be made by its manufacturer, is not guaranteed or endorsed by the publisher.

## Supplementary Material

Supplementary Figure 1 **(A–C)** Analysis of the mRNA expression of human S1PR1, S1PR2, and S1PR3 in TAD lesions (*n* = 10) and normal thoracic aorta (*n* = 6). **(D–F)** Analysis of the mRNA expression of the expression of mouse S1PR1, S1PR2, and S1PR3 in TAD lesions (*n* = 6) and control thoracic aorta (*n* = 6). **(G)** Representative immunoblot and the corresponding analysis of human S1PR3 in TAD lesions (*n* = 10) and normal thoracic aorta (*n* = 6). **(H)** Representative immunoblot and the corresponding analysis of mouse S1PR3 in TAD lesions (*n* = 6) and control thoracic aorta (*n* = 6).

Supplementary Figure 2 **(A)** Bodyweight in each group before angiotensin II infusion. **(B)** Systolic blood pressure in each group before angiotensin II infusion.

Supplementary Figure 3 A schematic diagram depicting the possible interaction relationship between S1PR2 signaling, immune cells, NETs, smooth muscle cells (SMCs), and the TAD formation.

Supplementary Table 1 Detail information of primers.
