# Supplementary figures and images for: Inhibition of Sphingosine-1-Phosphate Receptor 2 Prevents Thoracic Aortic Dissection and Rupture
Source: Front Cardiovasc Med. 2021 Dec 17;8:748486. doi: 10.3389/fcvm.2021.748486 (PMC8718435; doi:10.3389/fcvm.2021.748486)

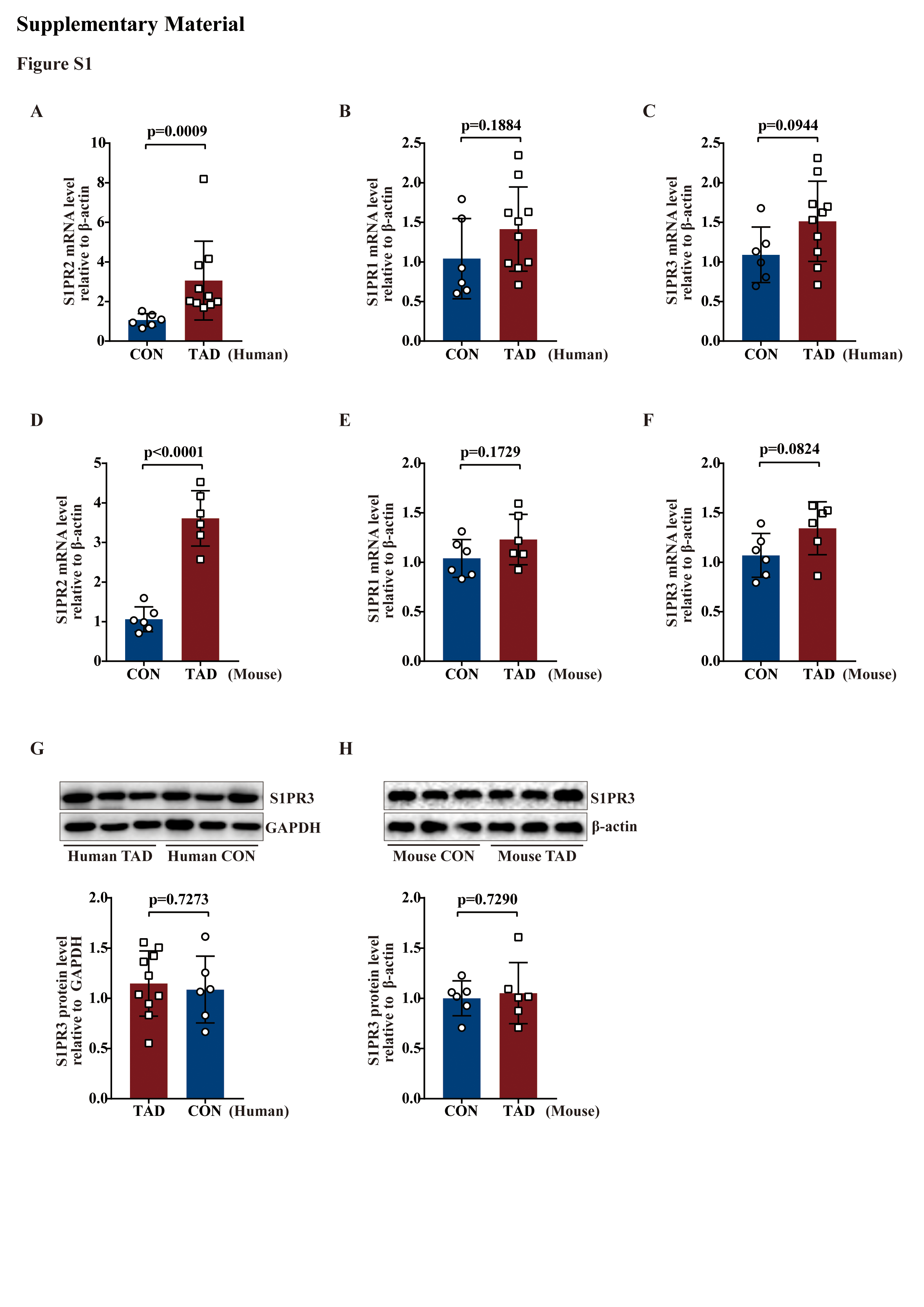

Supplement: Supplementary Figure 1 — (A–C) Analysis of the mRNA expression of human S1PR1, S1PR2, and S1PR3 in TAD lesions (n = 10) and normal thoracic aorta (n = 6). (D–F) Analysis of the mRNA expression of the expression of mouse S1PR1, S1PR2, and S1PR3 in TAD lesions (n = 6) and control thoracic aorta (n = 6). (G) Representative immunoblot and the corresponding analysis of human S1PR3 in TAD lesions (n = 10) and normal thoracic aorta (n = 6). (H) Representative immunoblot and the corresponding analysis of mouse S1PR3 in TAD lesions (n = 6) and control thoracic aorta (n = 6). [file Image_1.TIF]

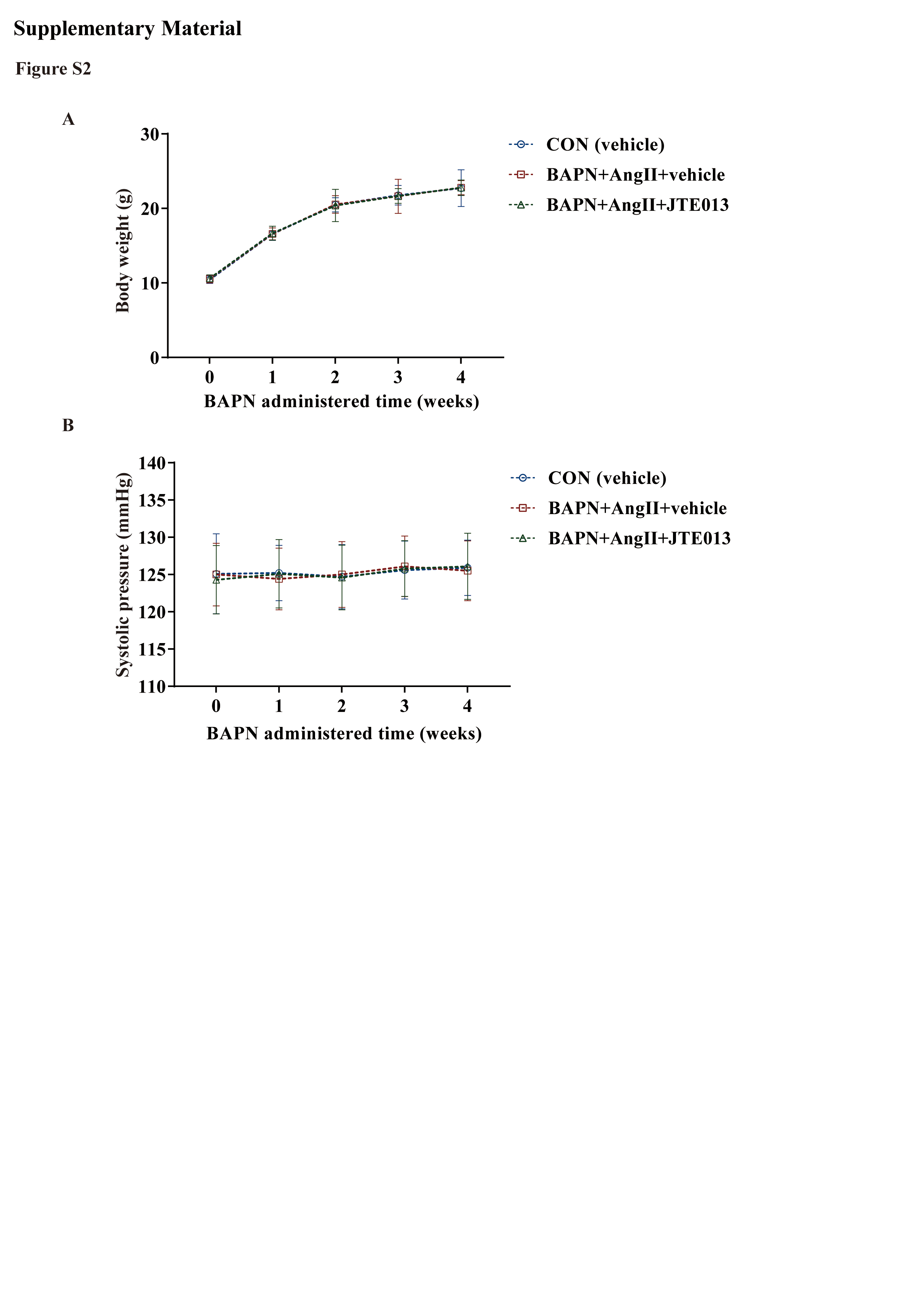

Supplement: Supplementary Figure 2 — (A) Bodyweight in each group before angiotensin II infusion. (B) Systolic blood pressure in each group before angiotensin II infusion. [file Image_2.TIF]

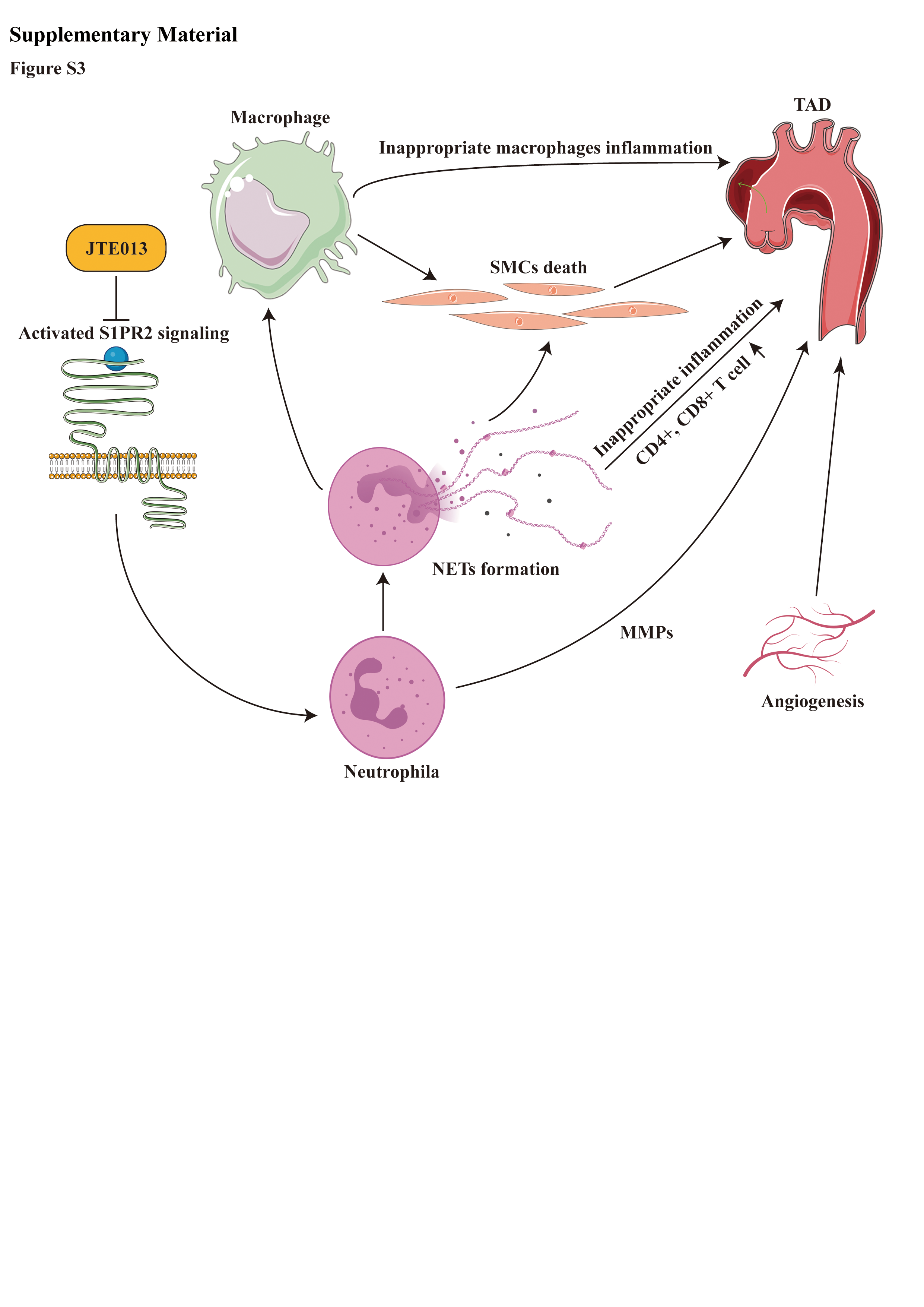

Supplement: Supplementary Figure 3 — A schematic diagram depicting the possible interaction relationship between S1PR2 signaling, immune cells, NETs, smooth muscle cells (SMCs), and the TAD formation. [file Image_3.TIF]
