# Supplementary material for: Inhibition of Sphingosine-1-Phosphate Receptor 2 Prevents Thoracic Aortic Dissection and Rupture
Source: Front Cardiovasc Med. 2021 Dec 17;8:748486. doi: 10.3389/fcvm.2021.748486 (PMC8718435; doi:10.3389/fcvm.2021.748486)
Supplement: Supplementary Table 1 — Detail information of primers. [file Table_3.DOCX]

**Supplementary Material**

**Table S1** Detail information of primers

| Gene |  | Sequence (5'-3') |
| --- | --- | --- |
| IL-1β | Forward | AGAAGATGGAAAAACGGTT |
|  | Reverse | CTTGTGCTCTGCTTGTGAG |
| IL-6 | Forward | GAAAATCTGCTCTGGTCTTCT |
|  | Reverse | CACTCCTTCTGTGACTCTAAC |
| MCP-1 | Forward | TTGAGGACAGACACAGCAGCC |
|  | Reverse | TCACCAATTCCCCTAGCACC |
| TNF-α  E-selectin  ICAM-1  VCAM-1  CXCL-2  CXCL-5  β-actin  S1PR1  S1PR2  S1PR3  MMP2  MMP9 | Forward  Reverse  Forward  Reverse  Forward  Reverse  Forward  Reverse  Forward  Reverse  Forward  Reverse  Forward  Reverse  Forward  Reverse  Forward  Reverse  Forward  Reverse  Forward  Reverse  Forward  Reverse | ATGTCTCAGCCTCTTCTCATTC  GCTTGTCACTCGAATTTTGAGA  ATGCCTCGCGCTTTCTCTC  GTAGTCCCGCTGACAGTATGC  CCATCACCGTGTATTCGTTTC  AGGTCCTTGCCTACTTGCT  TTGGGAGCCTCAACGGTACT  GCAATCGTTTTGTATTCAGGGGA  CCAACCACCAGGCTACAGG  GCGTCACACTCAAGCTCTG  GTTCCATCTCGCCATTCATGC  GCGGCTATGACTGAGGAAGG  GTGACGTTGACATCCGTAAAGA  GCCGGACTCATCGTACTCC  ATCATGGGCTGGAACTGCATCA  CGAGTCCTGACCAAGGAGTAGAT  CAGACGCTAGCCCTGCTCAAGA  TAGTGGGCTTTGTAGAGGA  ACAACCGCATGTACTTTTTCAT  TACTGCCCTCCCTGAGGAACCA  TTCAGCTCTGGGATGACCTT  CAAGGTGCTGGCTGAGTAGATC  TTGACAGCGACAAGAAGTGG  GCCATTCACGTCGTCCTTAT |
