# Supplementary material for: Inhibition of Sphingosine-1-Phosphate Receptor 2 Prevents Thoracic Aortic Dissection and Rupture
Source: Front Cardiovasc Med. 2021 Dec 17;8:748486. doi: 10.3389/fcvm.2021.748486 (PMC8718435; doi:10.3389/fcvm.2021.748486)
Supplement: Supplementary file 5 [file Table_1.DOCX]

**ABBREVIATIONS**

TAD: thoracic aortic dissection

S1PRs: sphingosine-1-phosphate receptors

BAPN: β-aminopropionitrile fumarate

SPHK1: sphingosine kinase 1

S1P: sphingosine-1-phosphate

NETs: neutrophil extracellular traps

SMCs: smooth muscle cells

AAA: abdominal aortic aneurysm

BMI: body mass index

DMSO: dimethyl sulfoxide

RT-qPCR: Real-Time quantitative polymerase chain reaction

CitH3: citrullinated histone H3

FPG: fasting plasma glucose

LVEF: left ventricular ejection fractions

SBP: systolic blood pressure

DBP: diastolic blood pressure

hs-CRP: high-sensitivity C-reactive protein

WBC: white blood cell

MPO: myeloperoxidase

SD: standard deviation

ANOVA: analysis of variance
