# Supplementary material for: Inhibition of Sphingosine-1-Phosphate Receptor 2 Prevents Thoracic Aortic Dissection and Rupture
Source: Front Cardiovasc Med. 2021 Dec 17;8:748486. doi: 10.3389/fcvm.2021.748486 (PMC8718435; doi:10.3389/fcvm.2021.748486)
Supplement: Supplementary file 6 [file Table_2.DOCX]

**Required materials download links**

**https://www.jianguoyun.com/p/DdcQw-IQu9XaCRjQmoQE**

**(Pass word: ABCDEFG)**
